# Masters theses from a university medical college: Publication in indexed scientific journals

**DOI:** 10.4103/0301-4738.60070

**Published:** 2010

**Authors:** Upreet Dhaliwal, Navjeevan Singh, Arati Bhatia

**Affiliations:** Department of Ophthalmology, University College of Medical Sciences and GTB Hospital, University of Delhi, Delhi - 95, India; 1 Department of Pathology, University College of Medical Sciences and GTB Hospital, University of Delhi, Delhi - 95, India

**Keywords:** Indexed journal, publication rate, postgraduate medical thesis

## Abstract

**Background:**

The thesis is an integral part of postgraduate medical education in India. Publication of the results of the thesis in an indexed journal is desirable; it validates the research and makes results available to researchers worldwide.

**Aims:**

To determine publication rates in indexed journals, of works derived from theses, and factors affecting publication.

**Settings and Design:**

Postgraduate theses submitted over a five-year period (2001-05) in a university medical college were analyzed in a retrospective, observational study.

**Materials and Methods:**

Data retrieved included name and gender of postgraduate student, names, department and hierarchy of supervisor and co-supervisor(s), year submitted, study design, sample size, and statistically significant difference between groups. To determine subsequent publication in an indexed journal, Medline search was performed up to December 2007.

**Statistical Analysis:**

Chi square test was used to compare publication rates based on categorical variables; Student's *t*-test was used to compare differences based on continuous variables.

**Results:**

One hundred and sixty theses were retrieved, forty-eight (30%) were published. Papers were published 8-74 (33.7 ± 17.33) months after thesis submission; the postgraduate student was first author in papers from 26 (54%) of the published theses. Gender of the student, department of origin, year of thesis submission, hierarchy of the supervisor, number and department of co-supervisors, and thesis characteristics did not influence publication rates.

**Conclusions:**

Rate of publication in indexed journals, of papers derived from postgraduate theses is 30%. In this study we were unable to identify factors that promote publication.

Writing a thesis is an essential requirement for the postgraduate medical degree in India.[1] It aims at development of a spirit of enquiry, and exposes the candidate to the techniques of research. In the long term, medical research improves the students' independent analytical problem-solving skills, and ability to critically interpret scientific literature.[2]

The next logical step in the process of research is to disseminate the results. Traditionally, this can be achieved either by presentation at a scientific meeting or publication in a peer-reviewed journal.[3] Presentations rapidly provide new information to conference attendees. However, this data is not available to the entire scientific community unless published in a reputed, widely circulated scientific journal.[3,4] In addition, publication in a peer-reviewed journal is the best test to judge the quality of the research.[5–8] Peer-reviewed publications derived from theses also inform the scientific community about the integration of teaching and research in medical education.[7]

Despite the acknowledged advantages of publishing, getting students to publish the results of their theses seems to be a global problem.[4,6–8] This study was conducted to determine the publication rate, and factors affecting it, of theses-related research conducted at a university medical college.

## Materials and Methods

After institutional ethical committee clearance, Masters theses submitted between 2001 and 2005 were retrieved from the college library archives. We estimated that a five-year period would provide enough data for our study. Theses submitted prior to 2001 were not included as the numbers of postgraduate students then were smaller, possible differences in methodology and institutional resources available at that time could have made the data incomparable with more recent theses. The year 2005 was chosen to allow at least two years for the later theses to reach publication.

Two authors (AB, UD) assessed each thesis; many different disciplines were included. The variables collected included the candidate's full name and gender; full title of the thesis; department; full name, department and hierarchy of the supervisor and co-supervisor(s); year of submission; study design (descriptive, observational, or experimental [(randomized controlled trial (RCT) or non-RCT experimental study)]; sample size; and statistically significant difference between groups, if any.

To determine subsequent full publication, a detailed computerized search of articles indexed by *Index Medicus* up to December 2007 was performed using the PubMed server. There are several reasons why we chose PubMed. There is a regional trend in favor of PubMed indexed journals. The Medical Council of India, in its guidelines for minimal qualifications for medical teachers recommends publication of research material in PubMed indexed national or international journals.[9] National ranking of medical colleges in India is also partly influenced by PubMed indexed publications issuing from that institution.[10]

Investigators suggest that using Medline and Embase together significantly improves the overall search coverage.[11] However, Embase is a paid service. Since the institutional library does not subscribe to it, we were unable to use it. We also searched for articles on the national database (IndMED). It contains articles from peer-reviewed Indian biomedical journals; one of its aims is to make available articles from journals that are not indexed with Medline. In our setup, articles published in these journals do not enjoy the same prestige as those indexed in PubMed. In local parlance, they are considered ‘non-indexed’. For this reason we did not include the data from IndMED in the final analysis. However, we did want to identify what proportion of theses was published in these journals.

Appropriate key words from the title combined with the candidate's name were used to identify the corresponding publication in both databases. In case no hit was obtained, the search was repeated using key words with the name of the supervisor, and again with each co-supervisor. A published manuscript was considered to be a derivative of the thesis when it satisfied both of the following criteria: 1) at least one of the authors of the thesis was an author of the publication, and 2) at least one of the outcomes from the thesis was an outcome of the publication. The number of papers published from a thesis, type of journal (national or international), month and year of publication, time lag to publication, and sequential location of the postgraduate student's name in the author byline were recorded.

The data from PubMed indexed articles were entered into an Excel spreadsheet and SPSS Version 13 was used for statistical analysis. Significance testing, using the Chi^2^ test and Fisher's exact test, was used to compare publication rates based on hierarchy, department, year, study design, and statistically significant difference between groups; Student's *t*-test was used to determine differences in publication rates based on number of co-supervisors. Since sample size did not follow a Gaussian distribution, the Mann-Whitney U test was used to compare sample size of theses that were published with those that were not.

## Results

One hundred and sixty theses, submitted between January 2001 and December 2005, were retrieved from the institutional library. Forty-eight (30%) were subsequently published in PubMed-indexed journals as 59 papers; the number of papers published from a single thesis varied from one to four [Table 1]. The postgraduate student was named first author in papers generated from 26 of the 48 (54%) published theses. When multiple papers were generated from a single thesis, the student was invariably first author in at least one of them [Table 1]. If there was a single publication (n = 39), the student was first author in only 17 (44%) papers, with the supervisor named first on the author byline in the rest.

**Table 1:** Number of publications per thesis

Number of publications per thesis	Number of thesis (%)	Number of papers	Position of student on author byline				
			
			Not named	First	Second	Third	fourth
1	39 (81)	39	2	17	15	4	1
2	8 (16)	16	-	12	2	2	-
4	1 (2)	4	-	3	1		

Of the published theses, 11 (23%) resulted in papers in national journals, 32 (67%) in international, and five (10%) were published in both national and international journals. The time period between thesis submission and publication of the first paper relating to it varied from eight to 74 months (mean 33.7 ± 17.33); one-third were published within the second year after submission [Table 2].

**Table 2:** Time lag between thesis submission and subsequent publication in an indexed journal

Time lag between thesis submission and publication (months)	Number of thesis (%)	Months: Cumulative number (%)
12	4 (8)	12:4 (8)
>12 to 24	16 (33)	24:20 (42)
>24 to 36	6 (13)	36:26 (54)
>36 to 48	12 (25)	48:38 (79)
>48 to 60	5 (10)	60:43 (90)
>60 to 72	4 (8)	72:47 (98)
>72	1 (2)	74:48 (100)

There were 102 male postgraduate students; 27 (26%) went on to publish papers from their theses in PubMed-indexed journals. Of the 58 female students, 21 (36%) published papers relating to their theses. This difference was not statistically significant (*P* = 0.213).

The year of submission (*P* = 0.242), department of origin of thesis (*P* = 0.521), hierarchy of the supervisor (*P* = 0.264), and number of co-supervisors (*P* = 0.431) did not significantly affect subsequent publication rates. In 48 theses (30%) the co-supervisors were from the same department as the supervisor, while in 107 (68.0%) some co-supervisors were from different departments. Thirty-seven of the latter theses (34%) were subsequently published in indexed journals as compared to the former (11; 23%). This difference was not statistically significant (*P* = 0.189).

There were eight descriptive, 63 observational and 89 experimental studies; 36 of the latter were randomized controlled trials. Design of study did not influence publication rates (*P* = 0.436). Sample size varied between 20 and 3000 (median 60). Publication rates did not vary significantly with sample size (*P* = 0.321). Results with statistically significant differences were published at the same rate as others (*P* = 0.265).

We retrieved four papers from IndMED that corresponded with four theses. Three theses were submitted to the University in 2001, and one in 2002. Author names had been misspelled in two of the four papers.

## Discussion

The primary purpose of the Masters thesis is to educate the candidate in scientific methods and to develop a scientific temper.[1] The results derived amount to scientific research and merit wide dissemination. Researchers have suggested that the real value of scientific work lies in its publication in indexed literature.[12] Publication makes research results visible and easily accessible to scientists anywhere in the world.[3] In addition, it enhances the academic and professional credibility of the researchers, as well as that of the department and of the institution.[13] However, many theses remain unpublished.[4,6–8]

In our setup, students ‘may be encouraged’ to publish thesis research in peer-reviewed journals; the university does not mandate it. The emphasis is on PubMed-indexed journals. However, two-thirds of the theses submitted over a five-year period in our institute were neither published nor were the results put up on the institutional or university websites; there is no policy in this regard. This scientific material, being available only to those researchers with access to the institutional library, is lost to the scientific community at large. A very small proportion of the earlier theses were published in national journals that are not indexed with PubMed (four theses, compared to 48 in PubMed). In the light of regional preferences, it is no surprise that the later theses were sent for publication to PubMed-indexed journals and not to IndMED-indexed ones.

There is no literature from India on the fate of theses-related research, so we reviewed the literature from smaller countries around the globe. Lack of publication of thesis-derived papers has also been reported from France, Finland and Croatia.[6,8,14] Low publication rate makes the quality of research suspect,[14] and compromises the quality of postgraduate medical education.[8,15] In addition, it represents a waste of manpower, money, and other resources.

Masters theses are neither expected to result in innovative research[4] nor should they be conducted simply to fulfill a degree requirement, choosing topics that are not publishable.[16] Since research publications are essential requirements for academic jobs and promotions in most parts of the world, topics should be selected keeping in view their publishable quotient; simple studies could be chosen over complex, multiple objective ones.[8,16]

Whereas most postgraduate theses were not published, more than one derivative work was published from nine (5.6%) theses. Multiple publications originating from a single research project may be repetitive publications representing scientific misconduct.[17] However, analysis of these abstracts revealed that all the publications from a single thesis represented different aspects of the whole and there was no misconduct.

First authorship issues may pose a dilemma when research work involves a postgraduate student and one or more faculty members.[18–20] At the commencement of the thesis the student rarely has the skills or knowledge necessary to conceptualize and design a study.[20] However, authorship credit is determined by degree of scientific or professional contribution.[19] The scale invariably tips in favor of the supervisor for first authorship.

Most of the publications from theses in our institution appeared in the year after completion of the postgraduation course. Other studies have also shown that publication only starts two years after course completion.[4] Authors from France found that 27% of theses appeared in print in the first year, nearly 50% after two years.[6] The delay in publication is a window of missed opportunity indicative of a malaise in the system of postgraduate medical education. Postgraduate medical education is supposed to be an initiation to research; the process is incomplete until the work is published in a peer-reviewed journal. Research not published can get outdated rapidly; similar work from other centers may be published in the interim. Even though this is common knowledge, very little is being done to encourage postgraduates to publish.[4,21]

Publication success has been linked to supervisors' supportive role in scientific publishing activity.[22,23] Supervisors have a responsibility as mentors to encourage postgraduates to publish, and to facilitate professional progression.[24] An experiment at two premier research institutes in India, where the thesis was removed from the postgraduate curriculum, resulted in a marked reduction of publications from those departments over that period.[24] This suggests that the thesis is an important resource for potentially enhancing the publication status of both supervisor and candidate. Interestingly, having published during the undergraduate period has been identified as a factor that encourages publication later.[22] Conversely, low publication rates may be related to high workload of researchers with teaching and routine professional obligations, financial constraints, and lack of sophisticated equipment.[8,22]

None of the factors that we studied had a significant role in encouraging publication of thesis results in peer-reviewed journals. This study was performed in the local setting of one medical college in India. However, the malaise is global, even though each institution is unique. Thus, the findings might be useful when considering interventions in postgraduate training. Researchers have found significantly different publication rates between two universities in the same country, suggesting different institutional emphasis directed towards publication.[14] Our institution has not specifically explored this avenue. Perhaps institutional endorsement, recognition and reward for published work may help. In the interim, to make theses-related research visible, medical institutions could publish thesis results on their web archives.[8]

In this study, we found that only 30% of theses-related research conducted in a University medical college is published in indexed, peer-reviewed journals. We were unable to identify factors that promote publication.
